# Expression of CXCR4 on CD4^+^ T cells predicts body composition parameters in female adolescents with anorexia nervosa

**DOI:** 10.3389/fpsyt.2022.960905

**Published:** 2022-09-26

**Authors:** Jana Freff, Lisa Bröker, Rafael Leite Dantas, Kathrin Schwarte, Judith Bühlmeier, Isabelle Kraft, Anke Hinney, Ulrike Buhlmann, Volker Arolt, Udo Dannlowski, Georg Romer, Bernhard T. Baune, Johannes Hebebrand, Manuel Föcker, Judith Alferink

**Affiliations:** ^1^Department of Psychiatry, University of Münster, Münster, Germany; ^2^Cells in Motion Interfaculty Cluster, University of Münster, Münster, Germany; ^3^Department of Child and Adolescent Psychiatry and Psychotherapy, University Hospital Essen, University of Duisburg-Essen, Essen, Germany; ^4^Department of Clinical Psychology and Psychotherapy, University of Münster, Münster, Germany; ^5^Faculty of Natural Sciences, Institute of Nutrition, Consumption and Health, University Paderborn, Paderborn, Germany; ^6^Institute for Translational Psychiatry, University of Münster, Münster, Germany; ^7^Department of Child and Adolescent Psychiatry and Psychotherapy, University Hospital Münster, Münster, Germany; ^8^Department of Psychiatry, University of Melbourne, Melbourne, VIC, Australia; ^9^The Florey Institute of Neuroscience and Mental Health, University of Melbourne, Melbourne, VIC, Australia

**Keywords:** anorexia nervosa, eating disorder, T cell, immune system, chemokine receptor, inflammation

## Abstract

Anorexia nervosa (AN) is a severe eating disorder characterized by excessive weight loss and lack of recognition of the seriousness of the current low body weight. Individuals with AN frequently exhibit an enhanced inflammatory state and altered blood levels of cytokines and chemokines. However, the expression of chemokine receptors in AN and the association with body composition parameters and treatment effects are still unknown. In this study, we examined the expression of CCR4, CCR6, CXCR3, and CXCR4 on peripheral blood T cells in female adolescents with AN before (T0, *n* = 24) and after 6 weeks of multimodal therapy (T1, *n* = 20). We also investigated their value to predict body mass index (BMI) and fat mass index (FMI) at baseline. Using multi-parameter flow cytometry, we found increased expression of CCR4, CXCR3, and CXCR4, but not CCR6, on CD4^+^ T cells in AN at T0 when compared to healthy controls (HC, *n* = 20). At T1, CXCR3 and CXCR4 expression decreased in AN. We found a close link between CCR4, CCR6 and CXCR4 expression and the adolescent mental health status in the study cohort as determined by the Strengths and Difficulties Questionnaire (SDQ). Specifically, CXCR4 expression correlated positively with emotional symptoms and peer relationship problems, as well as with the total sum score of the SDQ. In addition, CXCR4 expression on CD4^+^ T cells was a significant predictor of BMI and FMI in female adolescents. Our findings that CXCR4 expression on T cells is altered in adolescents with AN and predicts body composition parameters in adolescents suggest an impact of this chemokine receptor in the pathogenesis of AN.

## Introduction

Anorexia nervosa (AN) is a severe eating disorder with peak onset during adolescence and a high incidence in females (1–3). AN has the highest mortality rate among all mental disorders (4–7). Clinical symptoms of AN are characterized by low body weight, distorted body image, and weight phobia associated with restricted food intake and/or weight-controlling behaviors (8–11). Several body composition parameters such as body mass index (BMI) and fat mass (FM) are reduced in AN and related to the severity of malnutrition (12). We and others have shown before that the body composition parameters FM and fat free mass index (FFMI) are also associated with endocrine and immunometabolic adaptations (13–17).

In addition to genetic and environmental risk factors, the immune system is thought to play a pathophysiological role in AN (3, 18–25). A dysregulated immune system due to impaired eating behavior or malnutrition has been shown to impair the activation and function of immune cells, which may have negative effects on the defense against pathogens (22, 26). A bidirectional relationship between eating disorders and autoimmunity is well established (27–29), and genetic risk factors with genome-wide significance associated with immune responses have been identified (22, 23, 30). Changes in pro-inflammatory cytokines and numbers of T and B lymphocytes in the peripheral blood of individuals with AN point toward a pathophysiological involvement of the innate and adaptive immune system (22, 31–34). Accordingly, we recently demonstrated changes in B cell maturation stages in the peripheral blood of adolescents with AN and a strong association with fat mass index (FMI) and FFMI (13).

Chemokines are chemotactic proteins that control chemotaxis, activation and differentiation of immune cells by signaling through cell surface G-protein coupled chemokine receptors (35, 36). A bidirectional relationship between different chemokine-chemokine receptor axes and the secretion of leptin, a hormone that regulates appetite through a post-transcriptional mechanism, was found (37, 38). Recent studies provide insights into blood chemokine levels in AN with controversial results (24, 39–42). Dalton and colleagues found unchanged serum concentrations of several chemokines among those CCL2 (MCP-1), CCL3 (MIP-1α), CCL4 (MIP-1β), CCL11 (Eotaxin), CCL13 (MCP-4), CCL17 (TARC), and CCL26 (Eotaxin-3) in 23 subjects with AN compared to 13 HC (31). In a cross-sectional study, however, individuals with AN (*n* = 56) had lower blood CCL4 concentrations as HC (*n* = 51), and CCL4 levels correlated negatively with BMI and eating disorder psychopathology (43). A close association between chemokine levels and body parameters regarding intracellular, extracellular and total body water balance was also reported for CCL13 in individuals with AN and HC (44). However, knowledge is scarce about the expression profiles of chemokine receptors in AN and their association with body composition parameters.

The chemokine receptors CCR4, CCR6, CXCR3, and CXCR4 control homing and effector functions of T cells implicated in inflammatory responses and behavior (45–52). For example, CCR4 is a key regulator of neuroinflammation by promoting Th17 migration and modulates emotional and cognitive behavior in human and mice (51, 53–58). Also CCR6, the unique receptor for CCL20, mediates chemotaxis of leucocytes and has been functionally involved in emotion-like, cognition-like and sociability behaviors in mice (59–61). The CXC chemokine receptor CXCR3 controls CXCL9, CXCL10, and CXCL11 induced leucocyte trafficking to inflammatory sites (62, 63), and reduced CXCR3 expression on CD4^+^ T cells and CXCR3^+^ CD4^+^ T cell numbers have been found in mental disorders (64, 65). The CXCR4–CXCL12 axis crosstalks with several neurotransmitter systems and is part of the circuits controlling feeding behavior and metabolism (66). In the immune system, CXCR4 controls CXCL12 dependent migration of memory T cells to bone marrow niches which is required for their self-renewal (67). To date, the surface expression of these chemokine receptors and their potential as clinical predictors of body composition parameters in adolescence have not been studied.

In this exploratory study, we examined CCR4, CCR6, CXCR3 and CXCR4 expression on peripheral blood CD4^+^ T cells in female adolescents with AN and HC by multi-parameter flow cytometry, to detect state or trait related alterations. We investigated the association between receptor expression and mental health, body composition and weight related parameters. Finally, we analyzed the predictive impact of chemokine receptor expression on BMI and FMI in the study cohort.

## Materials and methods

### Subjects

Female patients with AN (*n* = 24) and healthy controls (HC, *n* = 20) were recruited at the Department of Child and Adolescent Psychiatry, Psychotherapy, and Psychosomatics, University Hospital Essen, University of Duisburg-Essen, Essen, Germany. For a more detailed description of the study group, the treatment regime performed, the anthropometric measurements and the collection of the blood samples, we refer to our recent publication (13). In the same study cohort, we reported earlier maturation stages of peripheral blood B cells in AN and HC (13). In brief, this study was conducted in accordance with the Declaration of Helsinki and approved by the Ethics Committee of the Medical Faculty of the University of Duisburg-Essen (12-5289-BO). All patients and controls and their parents gave written informed consent prior to the study. Inclusion criteria were female gender, age 12–18 years, and European ancestry. An additional inclusion criterion for the patient group was an AN diagnosis confirmed by clinical examination and semi structured interview, the Kiddie Schedule for Affective Disorders and Schizophrenia (K-SADS), according to the Diagnostic and Statistical Manual of Mental Disorders, fourth edition, text revision (DSM-IV-TR) (68). One patients’ BMI was at the 12th age- and sex adjusted BMI percentile, but as all other DSM-IV-TR criteria for AN were met, the patient was included in our study. Exclusion criteria for patients and HC were a severe comorbid mental disorder, alcohol or drug abuse, chronic endocrinological or inflammatory diseases, cancer, insufficient German language skills and IQ <70. Psychiatric disorders and severe somatic diseases were excluded based mainly on participants’ medical and psychiatric history. For assessment of emotional and behavioral items in childhood and adolescence the SDQ was applied in the whole study group (69). All participants were non-smokers (*n* = 44). All participants in the control group had regular menstrual cycles (*n* = 20), whereas 21 anorexic patients were amenorrheic. Three patients with AN were taking oral contraceptives and reported withdrawal bleeding. One patient with AN was receiving benzodiazepines and domperidone at T0. Three patients with AN were taking vitamin D because of vitamin D deficiency. None of the subjects in the control group were taking medication.

### Treatment

All patients with AN were admitted to psychiatric inpatient care. Treatment was based on the German S3 guideline with a multimodal, cognitive and dialectical-behavioral therapy concept (70, 71). A detailed description of the treatment concept can be found in Freff et al. (13).

### Screening variables

#### Anthropomorphic measurements

As described before, we performed anthropomorphic measurements at the acute state of starvation (T0) and after 6 weeks of inpatient treatment (T1). We conducted body weight, height, chest-, abdominal- and hip circumference measurements using earlier described standard operating procedures. In addition, body composition parameters such as FM assessed by the BodPod^®^, FMI, BMI, BMI standard deviation scores (BMI SDS) were determined. For details see Freff et al. (13).

#### Eating disorder examination questionnaire

Dysfunctional eating behavior was assessed using the German version of the Eating Disorder Examination Questionnaire (EDE-Q) (72) to screen the severity of the eating disorder. The EDE-Q is a widely used instrument to assess eating disorder specific psychopathology as well as diagnostically relevant core behaviors. It assesses eating disorder-specific characteristics using four subscales including (i) restrained eating, (ii) eating-related worries, (iii) weight worries, and (iv) figure worries (72). A total of 22 items are assigned to these subscales. Six additional items are used to record diagnostically relevant core behaviors, such as binge eating or self-induced vomiting. All items are to be assessed for the period of the last 28 days using seven-point rating scales according to frequency and intensity (0 = attribute non-existent; 6 = attribute existent every day/in an extreme degree) (72). High internal consistencies are shown for both the subscales as well as for the total score of this questionnaire procedure. Moreover, retest reliability, tested on a non-clinical sample, shows stability over a 3-month period (72).

#### Strengths and difficulties questionnaire

The SDQ (69) is used to measure symptoms of mental health disorders in 3–16 year olds. The questionnaire consists of 25 items comprising five subscales: (i) emotional symptoms (anxiety and depressive symptoms), (ii) behavioral problems, (iii) hyperactivity/inattention, (iv) relationship problems with peers, and (v) pro-social behavior (positive behaviors such as kindness and helpfulness, rated inversely to the other subscales). Response options are scored using a three-point rating scale (0 = strongly disagree, 1 = somewhat agree, 2 = strongly agree). The SDQ discriminates well between children and adolescents with and without psychopathology symptoms (73–75), and can be used as an effective screen for child/adolescent psychiatric disorders in the general population (76).

#### Blood sample collection, cryopreservation, and thawing of peripheral blood mononuclear cells

Blood sample collection was performed as described before (13). Briefly, 15 ml freshly drawn peripheral blood was obtained in sterile sodium heparin-treated tubes regularly from participants in the morning after an overnight fast. For isolation of peripheral blood mononuclear cells (PBMC), a standard density gradient centrifugation was performed according to the manufacturer’s instructions using Leucosep™ tubes (Frickenhausen, GER: Greiner Bio-One GmbH). Before cryopreservation of aliquots, isolated PBMC were washed three times with PBS supplemented with 2% fetal calf serum (FCS). Afterward, cells were overnight stored at −80°C and later transferred to the gas phase of liquid nitrogen until further use. For this, 1 × 10^7^ cells in X-Vivo 15™ (Basel, CH: Lonza Group Ltd) supplemented with 10% FCS were mixed 1:1 with freezing medium, which consists of 80% FCS and 20% dimethyl sulfoxide (DMSO). On the day of use, PBMC were carefully thawed in a 37°C water bath and immediately washed with PBS supplemented with 2% FCS.

#### Flow cytometry

The whole blood staining procedure for surface marker expression was performed similar as described before (77). In brief, for identification of T cells and their subsets, monoclonal antibodies anti-human CD3-PerCP, anti-human CD4-APC-Cy7, and anti-human CD8-BV510 were used. Chemokine receptor expression was analyzed using monoclonal antibodies anti-human CCR4-PE-Cy7, anti-human CCR6-FITC, anti-human CXCR3-APC, and anti-human CXCR4-PE. In brief, thawed PBMCs were stained for CCR4, CCR6, CXCR3, and CXCR4 at 37°C for 15 min. Antibodies for T cell staining (CD3, CD4, CD8) were directly added afterwards to the samples followed by another incubation step for 45 min at room temperature (RT), protected from light. All antibodies were purchased from BioLegend^®^ (San Diego, CA, USA). Samples were acquired on a CytoflexS^®^ flow cytometer (Krefeld, GER: Beckman Coulter GmbH) and analyzed by FlowJo™ Software v10 (Ashland, OR: Becton, Dickinson and Company; 2019).

#### Statistical procedure

Statistical analyses were performed using Statistical Package for the Social Sciences (SPSS) version 28. Data were visualized with GraphPad Prism version 9. For analyses of group differences in demographic data, *t*-tests were performed for continuous variables. For outcome measures, normal distribution of data within the groups was tested by means of Shapiro–Wilk Test. Possible baseline differences in outcome variables between patients (T0 or T1) and HC were calculated using unpaired *t*-tests. Differences within anorectic patients (T0 vs. T1) were evaluated using paired *t*-tests. For correlational studies, Spearman correlation coefficient was used. Furthermore, the influence of chemokine receptor expression on the variance of BMI and FMI was calculated by multivariate linear regression (MLR). *p < 0.05; ^**^p < 0.01; ^***^p < 0.001 were considered statistically significant.

## Results

Anthropometric and clinical characteristics of participants with AN and HC are summarized in Table 1. In accordance to the earlier published study protocol, all participants were female adolescents with AN of the restricting subtype (13). There was no significant difference (*p* = 0.248) in the mean age between the AN (15.6 ± 1.4 years) and HC group (16.1 ± 1.6). We found significantly lower body composition parameters such as BMI, BMI SDS, percentage FM, and FMI in adolescents with AN than in HC (Table 1). We determined mental health items such as emotional symptoms, conduct problems, hyperactivity, peer relationship problems, and pro-social behaviors by the SDQ. Compared with HC, the mean scores of the SDQ subscales emotional symptoms and peer relationship problems, and the SDQ sum score were higher in patients with AN when compared to HC (*p* < 0.001) (Table 1).

**TABLE 1:** Characteristics of the study sample.

	HC (*n* = 20)		AN pre-treatment (T0, *n* = 24)		
	Mean	± SD	Mean	± SD	*P*-value
Body height (cm)	169.13	± 6.68	165.45	± 5.12	**0.045**
Chest circumference (cm)	86.33	± 4.79	74.85	± 4.43	**<0.001**
Abdominal circumference (cm)	66.43	± 3.81	58.33	± 4.10	**<0.001**
Hip circumference (cm)	89.15	± 6.02	77.06	± 3.98	**<0.001**
BMI (kg/m^2^)	20.53	± 1.93	16.01	± 1.46	**<0.001**
BMI SDS (KIGGS)	−0.35	± 0.74	−2.64	± 1.47	**<0.001**
FM (%)	24.95^#^	± 5.57	16.97^+^	± 4.26	**<0.001**
FMI (kg/m^2^)	5.17^#^	± 1.47	2.76^+^	± 0.79	**<0.001**
SDQ emotional symptoms	2.35	± 2.08	6.67	± 1.66	**<0.001**
SDQ conduct problem	1.35	± 1.27	1.41	± 1.21	0.845
SDQ hyperactivity	2.65	± 2.13	3.46	± 1.98	0.200
SDQ peer relationship problem	1.55	± 0.94	3.17	± 1.81	**0.002**
SDQ pro-social	8.90	± 1.02	9.08	± 1.10	0.448
SDQ sum score	7.90	± 3.43	14.58	± 3.96	**<0.001**

^#^n = 19.

^+^n = 23.

HC, healthy control; AN, anorexia nervosa; BMI, body mass index; SDS, standard deviation scores; KIGGS, German health interview and examination survey for children and adolescents; FM, fat mass; FMI, fat mass index; SDQ, strengths and difficulties questionnaire.

Significant effects are in bold print.

Outcome measures including various body composition parameters before (T0) and after 6 weeks of multimodal treatment (T1) in the AN group are displayed in Table 2. Body composition parameters including chest-, abdominal- and hip circumference, BMI, BMI SDS, FM, and FMI were significantly increased at T1 compared to T0. We also compared the specific eating disorder psychopathology in the treated AN group to baseline measures at admission using the Eating Disorder Examination-Questionnaire (EDE-Q). Scores of EDE-Q subscales such as restraint, eating concern, and weight concern significantly decreased at T1 compared to scores measured at T0. In addition, the EDE-Q sum score in the AN group was lower after treatment (Table 2). These data indicate that multi-modal therapy was effective in the patient group in improving body parameters and health-related eating behavior.

**TABLE 2:** Adolescents with AN before and after 6 weeks of inpatient treatment.

	AN pre-treatment (T0, *n* = 20)		AN post-treatment (T1, *n* = 20)		
	Mean	± SD	Mean	± SD	*P*-value
Chest circumference (cm)	75.15	± 4.65	78.28	± 5.16	**<0.001**
Abdominal circumference (cm)	58.93	± 3.76	62.63	± 4.22	**<0.001**
Hip circumference (cm)	77.48	± 3.87	80.23	± 3.97	**<0.001**
BMI (kg/m^2^)	16.29	± 1.22	17.42	± 1.17	**<0.001**
BMI SDS (KIGGS)	−2.40	± 1.11	−1.70	± 0.82	**<0.001**
FM (%)	17.14	± 4.13	21.50	± 4.99	**<0.001**
FMI (kg/m^2^)	2.80	± 0.75	7.86	± 1.78	**<0.001**
EDE-Q restraint	3.93	± 1.06	2.56	± 1.70	**<0.001**
EDE-Q eating concern	3.15	± 1.07	2.47	± 1.46	**0.023**
EDE-Q weight concern	4.14	± 1.26	3.47	± 1.76	**0.047**
EDE-Q shape concern	4.51	± 1.18	4.43	± 1.46	0.763
EDE-Q sum score	3.93	± 1.03	3.23	± 1.41	**0.007**

AN, anorexia nervosa; BMI, body mass index; SDS, standard deviation scores; KIGGS, German health interview and examination survey for children and adolescents; FM, fat mass; FMI, fat mass index; EDE-Q, eating disorder examination questionnaire.

Significant effects are in bold print.

### Altered expression profiles of CCR4, CCR6, CXCR3, and CXCR4 on CD4^+^ T cells in anorexia nervosa

**FIGURE 1:**
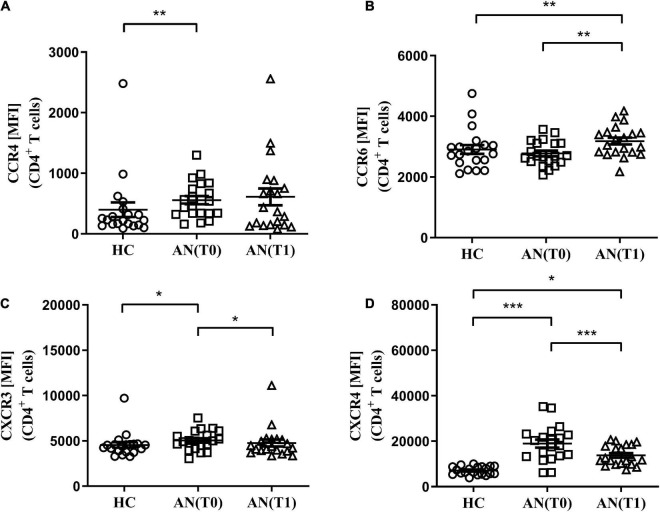
Mean fluorescence intensity (MFI) of chemokine receptors on CD4^+^ T cells. **(A)** CCR4, **(B)** CCR6, **(C)** CXCR3, and **(D)** CXCR4 MFI on CD4^+^ T cells in healthy controls (HC: *n* = 20), adolescents with anorexia nervosa (AN) at admission (T0, *n* = 20) and after 6 weeks of therapy (T1, *n* = 20), *p < 0.05; **p < 0.01; and ***p < 0.001.

We next examined the expression profile of CCR4, CCR6, CXCR3, and CXCR4 in adolescents with AN at T0 and T1, and HC using multi-parameter flow cytometry. In accordance with our previous findings, we found reduced frequencies of total blood lymphocytes in AN at T1 when compared to T0 and HC (Table 3) (13). We further determined lower CD3^+^ T cell frequencies in AN at T1 when compared to T0, while proportions of CD4^+^ and CD8^+^ T cells were not affected (Table 3). To characterize expression of chemokine receptors on CD4^+^ T cells, we measured mean fluorescence intensity (MFI) on the cellular surface of these cells. We found increased MFI for CCR4, CXCR3, and CXCR4, but not CCR6, on CD4^+^ T cells in AN at T0 compared to HC (Figures 1A–D and Table 3). At T1, CXCR3, and CXCR4 expression on CD4^+^ T cells decreased, while CXCR4 expression levels remained higher when compared to HC (Figures 1C,D and Table 3). CCR6 expression on CD4^+^ T cells, instead, increased at T1 when compared to T0 and HC (Figure 1B and Table 3). These results indicate that expression of these chemokine receptors on peripheral T cells is altered in female adolescents with AN when compared to HC.

**TABLE 3:** Comparison of immunological characteristics between HC and AN at T0 and T1.

	HC (*n* = 20)		AN pre-treatment (T0, *n* = 24)		AN post-treatment (T1, *n* = 20)		
			
	Mean	± SD	Mean	± SD	Mean	± SD	*P*-value
Lymphocytes (%)	69.12	± 5.92	70.97	± 6.36	65.35	± 8.32	0.231^a^ **0.012^b,c^**
CD3^+^ T cells (%)	62.02	± 7.44	61.43	± 10.07	57.30	± 10.13	0.888^a^ **0.010^b^** 0.125^c^
CD4^+^ T cells (%)	60.14	± 9.16	56.69	± 9.78	55.50	± 7.41	0.440^a^ 0.076^b^ 0.332^c^
CD8^+^ T cells (%)	27.85	± 6.76	27.33	± 6.81	27.39	± 7.45	0.619^a^ 0.379^b^ 0.782^c^
CCR4 (MFI) on CD4^+^ T cells	398.62	± 535.16	588.58	± 404.49	610.44	± 620.17	**0.003^a^** 0.823^b^ **0.461^c^**
CCR6 (MFI) on CD4^+^ T cells	2906.35	± 653.55	2797.79	± 425.76	3184.40	± 503.07	0.738^a^ **0.002^b^** **0.008^c^**
CXCR3 (MFI) on CD4^+^ T cells	4549.25	± 1354.69	4963.63	± 1097.06	4760.95	± 1707.82	**0.040^a,b^** 0.127^c^
CXCR4 (MFI) on CD4^+^ T cells	7097.50	± 1825.90	19049.79	± 7791.31	13771.35	± 4347.93	**<0.001^a,b^** **0.015^c^**

^a^HC vs. AN T0.

^b^AN T0 vs. AN T1.

^c^HC vs. AN T1.

HC, healthy control; AN, anorexia nervosa; MFI, mean fluorescence intensity.

Significant effects are in bold print.

### Close link between chemokine receptor expression and mental health problems

To examine the relationship between chemokine receptor expression on CD4^+^ T cells and adolescent mental health problems, we performed correlational studies (Table 4). Regarding CXCR3, no correlation was found. Expression of CCR6 on CD4^+^ T cells correlated negatively with hyperactivity (ρ = −0.357, *p* < 0.05) and the SDQ sum score (ρ = −0.315, *p* < 0.05). Furthermore, we found a positive correlation between CCR4 expression and emotional symptoms (ρ = 0.405, *p* < 0.01). In addition, CXCR4 expression correlated positively with emotional symptoms (ρ = 0.634, *p* < 0.001), peer relationship problems (ρ = 0.488, *p* = 0.001), and the SDQ sum score (ρ = 0.633, *p* < 0.001). These results demonstrate that CCR4, CCR6 and CXCR4 levels on blood CD4^+^ T cells in individuals with AN and HC closely correlate with specific mental health problems in adolescence.

**TABLE 4:** Correlation of immune parameters with psychological characteristics in AN and HC.

	CCR4 (MFI) on CD4^+^ T cells		CCR6 (MFI) on CD4^+^ T cells		CXCR3 (MFI) on CD4^+^ T cells		CXCR4 (MFI) on CD4^+^ T cells	
	
	ρ	*P*-value	ρ	*P*-value	ρ	*P*-value	ρ	*P*-value
SDQ emotional symptoms	**0.405**	**0.006**	−0.063	0.685	0.297	0.051	**0.634**	**<0.001**
SDQ conduct problem	−0.164	0.289	−0.152	0.326	−0.167	0.280	0.155	0.315
SDQ hyperactivity	−0.026	0.867	−**0.357**	**0.017**	0.007	0.966	0.216	0.159
SDQ peer problem	0.045	0.769	−0.223	0.145	−0.031	0.842	**0.488**	**0.001**
SDQ pro-social	0.079	0.611	0.169	0.272	0.268	0.078	0.053	0.730
SDQ sum score	0.179	0.245	−**0.315**	**0.037**	0.088	0.571	**0.663**	**<0.001**

n = 44 (HC: n = 20, AN: n = 24).

SDQ, strengths and difficulties questionnaire.

Spearman Correlation Coefficients; Conventions for effect size Cohen’s ρ: | ρ| = 0.10 small effect; | ρ| = 0.30 medium effect; | ρ| = 0.50 large effect.

Significant effects are in bold print.

### CXCR4 receptor expression on CD4^+^ T cells serves as predictor of body mass index and fat mass index

**FIGURE 2:**
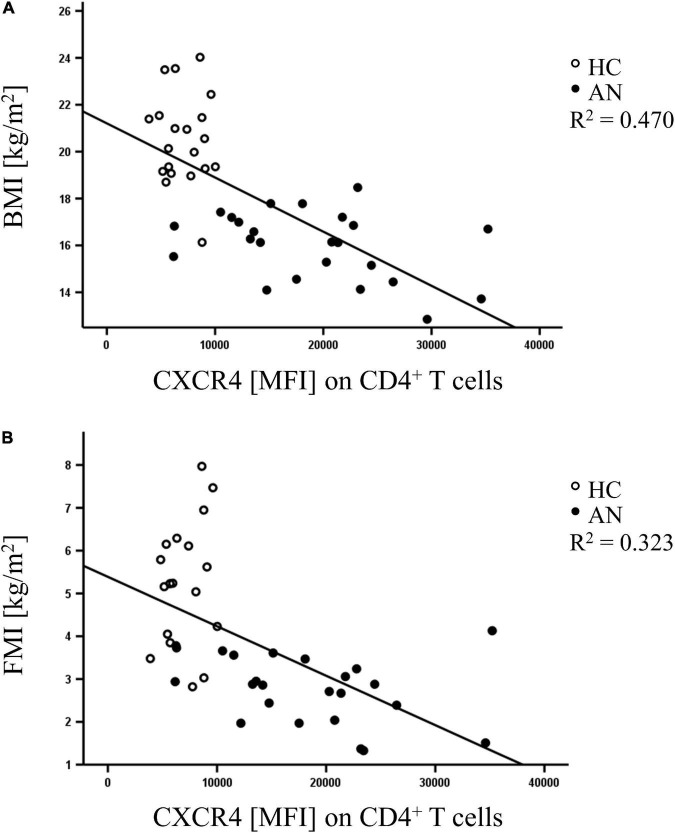
Scatter diagram depicting direct linear relationships between mean fluorescence intensity (MFI) of CXCR4 on CD4^+^ T cells and **(A)** body mass index (BMI), and **(B)** fat mass index (FMI) in healthy controls (HC: open circle, *n* = 20) and anorectic adolescents at admission (AN: black circle, *n* = 24).

We next examined whether receptor expression was associated with BMI and FMI in adolescents with AN at the acute stage of starvation and in HC. Therefore, MLR analysis was conducted using CCR4, CCR6, CXCR3 and CXCR4 expression on CD4^+^ T cells as independent variables to predict BMI and FMI of the participants of the study cohort (HC = 20; AN = 24). CXCR4 expression significantly predicted the participants’ BMI and explained 49.7% of variance. CXCR4 expression was also identified as significant predictor of FMI explaining 33.6% of variance (Table 5). In contrast, neither CCR4, CCR6, nor CXCR3 were predictors of body parameters. Furthermore, we found direct linear relationships between CXCR4 expression on CD4^+^ T cells and BMI (*R*^2^ = 0.470) and FMI (*R*^2^ = 0.323) in female adolescents (Figures 2A,B). These analyses demonstrate a close link between CXCR4 expression on blood CD4^+^ T cells and severity of AN and that CXCR4 expression is a clinical predictor of BMI and FMI in female adolescents.

**TABLE 5:** Multivariate linear regression analyses of BMI, FMI, and chemokine receptor expression.

Variable	B	SE	Beta	t	*P*-value
**BMI**					
(Constant)	24.307	2.493		9.749	<0.001
CCR4 (MFI) on CD4^+^ T cells	0.001	0.001	0.247	1.311	0.198
CCR6 (MFI) on CD4^+^ T cells	−0.001	0.001	−0.137	−0.892	0.378
CXCR3 (MFI) on CD4^+^ T cells	0.000	0.000	−0.132	−0.729	0.471
CXCR4 (MFI) on CD4^+^ T cells	0.000	0.000	−0.761	−5.991	**<0.001**
*F* = 9.628; df = (4,39); *p* ≤ **0.001**; *R* = 0.705; *R*^2^ = 0.497					
**FMI**					
(Constant)	6.506	1.769		3.677	<0.001
CCR4 (MFI) on CD4^+^ T cells	0.001	0.001	0.150	0.678	0.502
CCR6 (MFI) on CD4^+^ T cells	0.000	0.001	−0.118	−0.655	0.516
CXCR3 (MFI) on CD4^+^ T cells	0.000	0.000	−0.028	−0.132	0.895
CXCR4 (MFI) on CD4^+^ T cells	0.000	0.000	−0.627	−4.138	**<0.001**
*F* = 4.675; df = (4,37); *p* = **0.004**; *R* = 0.579; *R*^2^ = 0.336					

n = 44 (HC: n = 20, AN: n = 24).

BMI, body mass index; FMI, fat mass index; MFI, mean fluorescence intensity. Significant effects are in bold print.

## Discussion

Alterations of the immune system such as altered chemokine levels in the blood have been found in individuals with AN (22, 31). However, knowledge on the expression of chemokine receptors on T cells in adolescents with AN is scarce and neither treatment effects, nor associations with body composition parameters and mental health items have been studied. In this exploratory study, we determined cell surface levels of CCR4, CCR6, CXCR3, and CXCR4 on blood CD4^+^ T cells and identified CXCR4 as significant predictor of body composition parameters in adolescents with AN and HC.

Several studies investigated the effect of severe starvation on numbers of lymphocytes and their subsets in peripheral blood and found relative lymphocytosis or lymphopenia (33, 78, 79). Depending on the study design, decreased, equivalent or increased CD4^+^ T cell counts have been found in AN associated with altered CD4/CD8 ratios (33, 34, 79–81). In our study cohort, we found unaltered CD4^+^ and CD8^+^ T cell proportions in female adolescents with AN compared to HC. The observed decrease in blood CD3^+^ T cells in AN after therapy compared to inpatient admission may be a consequence of altered blood glucocorticoids in AN known to induce lymphocyte apoptosis or a consequence of hypoleptinemia during starvation causing multiple immune system alterations (14, 22, 82).

An important finding of our study was that surface expression of CCR4, CXCR3, and CXCR4 was increased on blood CD4^+^ T cells in adolescents with AN. To the best of our knowledge, this is the first study investigating the expression of these chemokine receptors in AN. In this context, appetite-regulating properties have been described for chemokine receptors and their ligands. For example, expression of CXCL12 has been found in the lateral hypothalamus of the adult brain, and expression of its cognate receptor, CXCR4, co-localized with melanin-concentrating hormone (MCH)-expressing neurons that regulate feeding behavior (66). In accordance, intracerebral administration of chemokines (CCL2, CCL3, CCL4, CCL5, CXCL4, CXCL8, and CXCL10) has been found to decrease food intake in rodents (83). In addition to the appetite-regulating capacities of chemokines and their receptors, they are also associated with the regulation of water distribution in the organism. For example, CXCL12 has been found to modulate the firing pattern of arginine-vasopressin neurons through CXCR4. It thus counteracts induced release of arginine-vasopressin that plays an important role in water and electrolyte balance (84). Interestingly, Himmerich and colleagues reported an association between CCL13 levels in peripheral blood and intra- and extracellular water balance in individuals with AN (44).

The molecular mechanism of upregulated receptor expression in AN observed in our study and its functional role in CD4^+^ T cells is still unresolved. The expression levels of chemokine receptors on human CD4^+^ T cells are tightly regulated by activating factors such as cytokines, chemokines, and corticosteroids (46). For example, IL-15 has been shown before to increase CCR4 and CXCR3 expression on blood CD4^+^ T cells *in vitro* (85). Therefore, elevated IL-15 concentrations demonstrated in AN (44, 86) could cause an increase in CCR4 and CXCR3 expression on blood T cells in the AN group of our study cohort. Other studies in AN demonstrated decreased blood levels of CXCL9, one of the ligands of CXCR3, and a correlation with the weight-regulating hormone leptin (41). Thus, upregulated CXCR3 expression on blood T cells in AN as observed in our study may also comprise a compensatory mechanism due to decreased CXCL9 serum levels in AN. It is well established that CXCR4 expression on T cells is tightly controlled by endogenous glucocorticoid receptor signaling (87), and cortisol has been shown to upregulate CXCR4 expression on CD4^+^ T cells in humans (88). In accordance, hypercortisolism has been determined in urine, plasma and saliva samples from AN subjects and found to be associated with lower hair cortisol concentration as markers for a dysregulated hypothalamic-pituitary-adrenal-axis in this eating disorder (89). Nevertheless, altered expression levels of chemokine receptors may not be a consequence of nutrient deficiencies. Although women with AN consume less dietary zinc and cholesterol than HC, nutrient intake patterns do not contribute significantly to altered concentrations of immune markers in the blood in AN (90).

Upregulation of surface chemokine receptor levels on CD4^+^ T cells in AN may alter adaptive immune responses. For example, CXCR4 drives migration of memory T cells from the blood to the bone marrow for self-renewal in response to CXCL12 (67). Increased CXCR4 expression on CD4^+^ T cells in AN may therefore alter sequestration of these cells in the bone marrow and negatively affect the immune response. Regarding CCR4, increased expression in AN on CD4^+^ T cells, which may represent Th17 cells, could promote autoimmunity and chronic inflammation (51, 55). Increased CCR4 expression in AN on regulatory CD4^+^ T cells, instead, may enhance their immunosuppressive functions and thus affect immune responses required in host defense (52, 53). However, chemokine receptor expressing T cells have not been further characterized in this study and future research is warranted to define their subtype in AN.

After refeeding during 6 weeks of multimodal therapy, CXCR3 levels normalized on CD4^+^ T cells in AN suggesting that this immune alteration is linked to the pathophysiology of this eating disorder. CXCR4 expression at T1 was also reduced but maintained at higher levels on CD4^+^ T cells when compared to HC, possibly due to incomplete weight recovery after therapy or additional stress factors due to refeeding. In the whole study sample, expression of chemokine receptors was significantly associated with social and behavioral problems as determined by SDQ scores. CXCR4 expression on T cells, in particular, correlated positively with emotional problems and peer problems in adolescence. While the cause of this close link is yet unexplained, CXCR4 expression has been found to be highly responsive to changes in the microenvironment and up-regulation of CXCR4 occurred in cell lines subjected to stress conditions such as growth factor deprivation, hypoxia, and space constraints (91).

Because off-label treatment of patients with AN with metreleptin has been associated with a rapid and prominent amelioration of emotional, cognitive and behavioral symptoms of this eating disorder (92, 93), it is tempting to speculate that one of the underlying mechanisms may relate to a metreleptin induced down-regulation of CXCR4. Indeed, it has been shown that caloric restriction alone increased the expression of CXCR4 protein in bone marrow derived mesenchymal stem cells, while co-culturing these cells with leptin following nutrient restriction decreased CXCR4 levels (94).

A prominent finding of our study was that expression of CXCR4 on CD4^+^ T cells significantly predicted BMI and FMI in female adolescents. CXCR4 expression levels on CD4^+^ T cells may therefore be used as a predictive clinical marker in future studies regarding therapy response in AN at admission since the body composition parameters BMI and FMI have been linked before to severity of AN (12, 95).

A limitation of this exploratory study is the small sample size that precludes generalizability of the results. Future studies with larger cohorts are needed to investigate the functional role of blood T cells that express chemokine receptors in AN and their value as clinical predictors for the course and severity of AN. To better understand the specific effects of CXCR4 on immune dysregulation in AN, it is also important to determine the Th17- and Treg-associated CXCR4 expression profiles, and the effector and regulatory abilities of those T cell subsets. In addition, the influence of adipokines, such as leptin, needs to be co-assessed in future studies to disentangle potential moderators and mediators.

Several immune and neuroendocrine factors such as IL-6, leptin, and oxytocin, as well as brain function and gut microbiome have been proposed as potential biomarkers to assess the severity and disease course of AN (96). Therefore, our study complements this active area of research by demonstrating that measurement of CXCR4 expression levels on peripheral blood T cells can be used as a rapid assay and as predictive marker for assessing the severity of AN.

In conclusion, our study demonstrates altered chemokine receptor expression in AN, and that the level of receptor expression on CD4^+^ T cells is associated with adolescent health problems. Upregulation of CXCR4 on blood T cells serves as clinical predictor for BMI and FMI in adolescence and may therefore be associated with pathophysiology of AN.

## Data availability statement

The raw data supporting the conclusions of this article will be made available by the authors, without undue reservation.

## Ethics statement

The studies involving human participants were reviewed and approved by the Ethics Committee of the Medical Faculty of the University of Duisburg-Essen. Written informed consent to participate in this study was provided by the participants’ legal guardian/next of kin.

## Author contributions

MF and JA: conceptualization. JF, KS, JB, IK, AH, MF, and JA: methodology. JF, LB, JB, IK, MF, and JA: formal analysis. JF, KS, JB, IK, MF, and JA: investigation. JF, LB, MF, and JA: writing – original draft preparation. RL, AH, UB, VA, UD, GR, BB, JH, MF, and JA: writing – review and editing. JF, LB, and JA: visualization. AH, MF, and JA: supervision. JA: funding acquisition. All authors reviewed the manuscript.
